# Patient navigation in German emergency care (NODE). Study protocol of a multi-center mixed-methods trial

**DOI:** 10.3389/frhs.2025.1524552

**Published:** 2025-08-26

**Authors:** Daniela Krüger, Maria Altendorf, Felix Holzinger, Cornelia Wäscher, Hanna Winkler, Martin Möckel, Christoph Heintze, Judith Stumm, Konrad Schmidt, Anna Slagman

**Affiliations:** ^1^ Department of Emergency and Acute Medicine (CVK, CCM), Charité – Universitätsmedizin Berlin, Berlin, Germany; ^2^ Institute of General Practice and Family Medicine, Charité – Universitätsmedizin Berlin, Berlin, Germany; ^3^ Institute of Social Medicine, Epidemiology and Health Economics, Charité – Universitätsmedizin Berlin, Berlin, Germany; ^4^ Institute of Medical Sociology and Rehabilitation Science, Charité – Universitätsmedizin Berlin, Berlin, Germany; ^5^ Institute of General Practice, Brandenburg Medical School Theodor Fontane, Brandenburg, Germany

**Keywords:** health care reform, patient navigation, emergency care, outpatient care, emergency department (ED), low-acuity, cross-sector cooperation, mixed-methods

## Abstract

**Background & objective:**

The objective of the study is to explore and to evaluate existing patient navigation models between emergency departments (ED) and alternative outpatient emergency care providers. Therefore, three different patient navigation models in emergency care in Berlin, Germany will be evaluated regarding their efficiency, quality of care, patient safety, patient satisfaction, costs and cost-effectiveness: (1) a hospital-owned separate outpatient care model within the ED premises or on the hospital grounds, (2) an urgent care practice of the Regional Association of Statutory Health Insurance (SHI) Physicians at the ED location, and (3) a standard care ED with prospective assessment of patient-care and urgency levels by ED doctors.

**Methods:**

In a convergent, parallel, mixed-methods, multi-center study design, patients' and health care providers' perspectives assessed by qualitative methods will be triangulated with the results of a quantitative prospective cohort study at six EDs in Berlin, Germany. This study includes four working modules: (1) a systematic literature review; (2) qualitative semi-structured interviews with patients (*n*∼15–20), as well as four focus group interviews with health care providers (*n*∼5); (3) a prospective multi-centre cohort study in adult, self-referred emergency patients at two time points (t0: *n* = 1,031; t1: *n* = 344), primary endpoint: proportion of patients treated other than standard emergency care, secondary endpoints: secondary efficiency, patient-reported outcome measures (PROMs), patient safety, quality of care, clinical outcomes, and cost-effectiveness; participant observation of patients' treatment trajectories and organizational processes at three different patient navigation models (*n*∼10–15), an online provider survey; and (4) data triangulation and recommendations for practice and policy.

**Discussion:**

This is the first study to compare different patient navigation and cooperation models to standard ED care in Germany and evaluate their effectiveness. Results will provide deep insights into the efficiency, patient satisfaction, quality of care, patient safety and cost-effectiveness. This will help providers and policymakers organise future emergency care in Germany. The study is expected to contribute to health services and systems research.

**Trial registration:**

DRKS00030398.

## 1 Introduction

Emergency departments (ED) are regularly frequented by patients with low-acuity consultation needs, estimated at about 20% to 33% (1–9). Taking into account the indicated level of medical care required and outpatient-specific needs (e.g., prescriptions, sick notes, long-term care), patients with low-acuity consultation needs could be treated appropriately and more cost-effectively in ambulant care settings (10). For the ED, low-acuity consultations are associated with crowding and pose challenges on health care systems and provision internationally (11, 12). Crowding is associated with adverse effects on the quality of care, patient safety, patient satisfaction, workload, and healthcare costs, as well as job satisfaction of medical staff (13, 14).

The German health care system does not operate a mandatory gatekeeper system. While this healthcare system's openness strengthens patients' choice and allows access to multiple health care providers, including any health care provider in an acute health situation, its institutional structure has also been described as contributing to ineffective care (15). Emergency and urgent care in Germany is offered by three main providers characterized by sectoral division, those being hospital EDs, the Regional Association of SHI physicians' in outpatient practices (during regular practice hours) including their on-call service (outside of regular practice hours), and the Emergency Medical Service (EMS), a public pre-hospital ambulance service. The sectoral divide between urgent and emergency care offered in ambulatory practices and hospital EDs impedes patient coordination and institutional cooperation.

Against the backdrop of persistently high patient numbers in EDs and the need for cross-sector cooperation, emergency care in Germany has recently undergone a period of local restructuring aimed at optimizing care pathways of patients: Urgent care practices operated with the SHI medical on-call service have been established in several locations, sometimes in proximity to or connected with an ED. In these care models, an initial assessment of urgency and suitability for an alternative ambulatory care setting is conducted. If an urgent care practice by the SHI is located near an ED (e.g., in the same building), cooperation is sometimes facilitated by a joint counter. There, the initial assessment is conducted and patients are assigned to either the ED or the SHI urgent care practice (16, 17). Low-acuity patients can be referred from the ED to SHI urgent care practices, and vice versa, resulting in different care pathways.

Internationally, such ED-integrated outpatient care units have been proven effective in reducing numbers of self-referred low-acuity patients in EDs (18). In Germany, several approaches are currently being discussed and tested regarding efficiency and patient safety (19). However, there is no standardized patient navigation pathway to direct self-referred patients presenting to the ED to the most appropriate health care provider. Instead, locally diverse and exclusively non-evaluated instruments and models are used to identify and re-direct patients who appear eligible for management in an urgent care model (20). A major associated concern is the current lack of evidence with regard to patient safety for such navigation and outpatient care models.

In this study, patient navigation is operationally defined as the diversion of patients between the emergency department and alternative care pathways. Navigation models differ in key aspects, including the timing of diversion, the criteria and methods used to assess patient suitability, the professional backgrounds of those involved in delivering care, and the extent to which the alternative care paths are integrated into existing ED workflows and physical structure. From a health systems perspective, the interplay between partially integrated and cooperative care structures is of particular relevance. This encompasses the development of shared organizational procedures and understandings, and the establishment of reliable, cross-sectoral collaboration between professionals from different healthcare sectors.

To date, no systematic overview of the currently practised patient navigation models in emergency care exists. The aim of this study is to systematically explore and evaluate existing patient navigation models in emergency care. Therefore, four working modules are included into the study: (1) a systematic literature review, (2) qualitative semi-structured interviews with patients, as well as focus group interviews with health care providers, (3) a prospective multi-centre cohort study in adult, self-referred emergency patients as well as participant observation of patients' treatment trajectories and organizational processes at three different patient navigation models, and an online provider survey, and (4) data triangulation, theory-informed synthesis, and recommendations for practice and policy.

The following research questions will be addressed within different working modules of this study:

1. Which patient navigation models in emergency care entailing navigating of eligible patients to an alternative care pathway or care setting are practised internationally? (module 1)
2. What is the evidence for these models in terms of quality of care (care process or outcome quality, incl. patient satisfaction), patient safety, and cost-effectiveness? (module 1)
3. Which patient navigation models in emergency care are practised in the pilot region of Berlin and in Germany? (module 2 & module 3)
4. How do providers and patients qualitatively evaluate different patient navigation models in emergency care that are already practised in Berlin? (module 2 & module 3)
5. How do different patient navigation models in emergency care practised in Berlin differ quantitatively in terms of efficiency, quality of care, patient safety, patient satisfaction, and cost-effectiveness? (module 3)
6. Which urgency and level of care show patients assessed by ED doctors in standard care EDs included in the study? (module 3)
7. How do the three included patient navigation models differ in terms of patient processing and care pathways, as well as patient experience (module 2 & module 3)?
8. What recommendations can be derived for political decision-makers from the findings of questions 1–7 for future organisation of patient navigation models in emergency care? (module 4)

## 2 Methods and analysis

### 2.1 Study design

A convergent, parallel, mixed-methods trial consisting of four working modules carried out in parallel, described in detail below, will be conducted. A mixed-methods approach is chosen to compare, integrate and validate the research results through triangulation for inclusion of different data sources, data methods and theoretical perspectives (21, 22).

**Figure 1:**
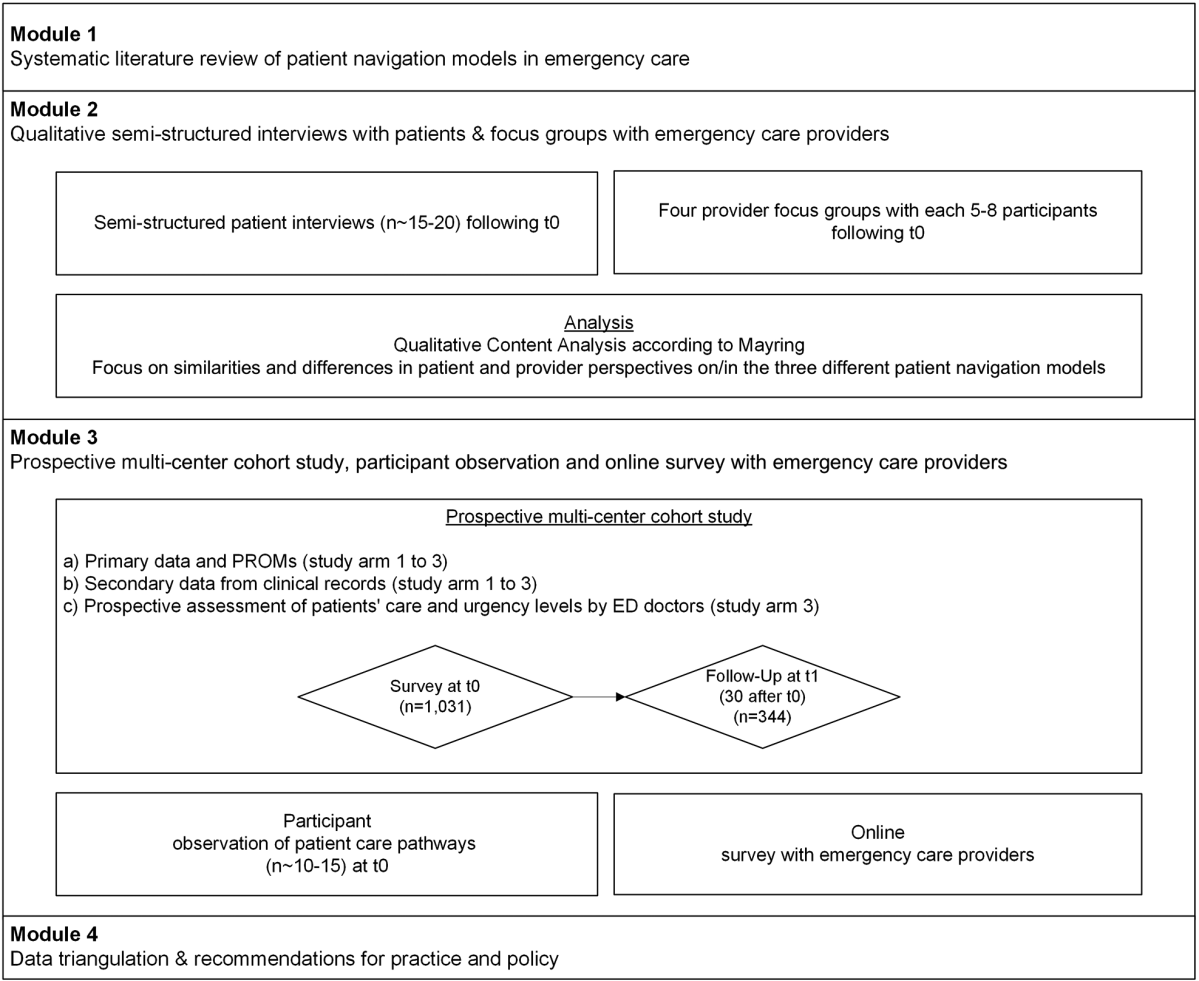
Study design of the NODE study. T0 = baseline, t1 = follow up. Study arm 1 = hospital-owned separate outpatient care model within the ED premises or on the hospital grounds; study arm 2 = urgent care practice of the Regional Association of SHI Physicians at the ED location; study arm 3 = ED with standard care model and prospective assessment of patient-care and urgency levels by ED doctors.

The total duration of the research project is set at 36 months, of which the data collection phase in the different modules and the data analysis will take 25 months. Data collection in module 2, which consists of qualitative interviews with patients and focus groups with providers, is set to a period of 21 months. Recruitment of participants for module 2 began on 6/1/2023 and will end on 02/28/2025. Data collection in module 3, the prospective multi-centre cohort study, participant observation and the provider survey, is set to a period of 22 months, including the extraction of routine data from clinical systems. Participant recruitment for the prospective multi-centre cohort study in module 3 began on 6/1/2023 and will end on 8/31/2024. Participant recruitment for the provider survey began on 7/1/2023 and will end on 7/1/2024. The study design with the different modules is illustrated in Figure 1.

### 2.2 Study arms

In module 2 and 3, three study arms will be compared:

(1) a hospital-owned separate outpatient care model within the ED premises or on the hospital grounds,
(2) an urgent care practice of the Regional Association of SHI Physicians at the ED location, and
(3) an ED with standard care model and prospective assessment of patient-care and urgency levels by ED doctors.

### 2.3 Modules

#### 2.3.1 Module 1: systematic literature review

In module 1, a systematic literature search, evidence synthesis and assessment will be conducted. Available studies will be searched, screened and categorised. Details on inclusion criteria (participants, interventions, study designs, outcomes), the screening process, data extraction, quality appraisal and data synthesis for the literature review has been described in a detailed study protocol published in the PROSPERO register (23). Results will be reported according to the PRISMA statement (24).

Since modules 2 and 3 share the same criteria for recruitment, sampling, and inclusion and exclusion criteria, these aspects will be described together for both modules before addressing the individual modules.

#### 2.3.2 Module 2 and 3: recruitment and sampling

Patients will be recruited in the waiting room of the ED or at a joint point of contact between the ED and an alternative care structure. During the consent process, patients will be informed about the study and invited to participate, and written consent is obtained. Patients participating in the prospective, quantitative survey module 3, will also be invited to participate in participant observation (if applicable on the recruitment day) and in qualitative, semi-structured interviews (module 2) at intervals after treatment. Thus, the qualitative samples for participant observation and semi-structured interviews are a subsample of the quantitative patient sample from module 3. Approximately 10–15 participant observations and 15–20 semi-structured interviews are planned. The exact number of interviews will be determined by theoretical saturation; the number of participant observations is based on the differences in study arms and centers and potential patient treatment paths. Recruitment of emergency care providers for all focus groups in module 2, and the online survey in module 3 (see below) will take place via established networks of the consortium partners and the relevant professional associations.

##### 2.3.2.1 Module 2 and 3: inclusion and exclusion criteria

Patients eligible for the study will meet to the following criteria:

– Age ≥18,
– Self-referred walk-in presenting to the ED.

Patients who are excluded from study participation present:

1. directly and intentionally to the alternative outpatient care unit,
2. have an insufficient knowledge of German or English language for written informed consent and study participation, and/or
3. have an insufficient cognitive status (e.g., patient being unable to give informed consent), and/or
4. are in need of immediate medical intervention in the ED and are thus not eligible for patient navigation.

Eligible participants for semi-structured interviews will be patients who have been treated within one of the study arms. Participants will be selected based on age, gender, highest level of education attained, any migrant background, number of chronic pre-existing conditions and the cooperation model used to represent the whole sample in module 3. Focus group participants will be physicians, nurses and administrative staff working in either a participating ED or in one of the study alternative care structures. Focus group participants will also purposively be selected by considering the heterogeneous representation of age, gender, specialisation and professional experience. The composition of the focus groups will be interdisciplinary and cross-study-arms. Eligible participants for participant observation in module 3 will be purposively selected to represent organizational differences within and between the study arms and different care pathways to complement patient interviews and focus groups in module 2.

#### 2.3.3 Module 2: qualitative prospective assessment

Patients and health care providers at six Berlin study sites will be evaluated: Patients (approximately *n* = 15–20) will be invited to participate in individual, semi-structured interviews either online or in person. Health care providers working in the participating EDs or an alternative patient navigation model in emergency care will be invited to participate in interdisciplinary focus group interviews (4 focus groups with each *n* = 5–8 participants). Semi-structured interviews have been chosen to ensure in-depth discussions of the topic. Interviews aim to explore the patients' perspective on emergency care, preferences regarding the patient navigation models in emergency care and care providers, perceived deficits of care and information, and recommendations to improve current emergency treatment. By means of focus groups, it is intended to deepen the understanding of health care providers' perspective by a dynamic exchange of perspectives and needs among different health care professions on different patient navigation models in emergency care with regards to efficiency, patient satisfaction, quality of care, patient safety, and costs. Moreover, health care providers will be asked to relate ideas for potential improvement of the different patient navigation models in emergency care. Interview and focus group guidelines will be developed in an iterative process based on studies from the previous literature, as well as on the experience of the research group.

##### 2.3.3.1 Data analysis plan of module 2

Semi-structured individual interviews and focus groups will be audio-recorded and transcribed verbatim. The transcripts will be pseudonymized and analyzed based on the content analysis according to Mayring (25), combining an inductive and a deductive approach. The deductively developed categories will be derived from both the literature and the researcher's prior knowledge. In addition, categories will be generated inductively from the transcripts. Within the framework of qualitative content analysis, the following procedural steps will be applied: (1) the material to be analyzed is determined, (2) analysis and documentation of the situation in which the material was created, (3) setting transcription rules, (4) determination of the interpretation focus, (5) theoretical justification of the research question, (6) determination of the analysis technique (summarized, explicative, structural or a combination), (7) determination of the analysis unit, (8) coding the text material, (9) categorization of the remaining material after reviewing the material and the research question, (10) interpretation. Controversial results are discussed in the research team until consensus is established (26). A particular focus during the analysis is placed on the patient-provider relationship, analyzing congruencies and incongruences between the patient and provider perspectives. The results will be reported in accordance with the standards for reporting qualitative research guideline (27).

#### 2.3.4 Module 3: prospective multi-center cohort study, participant observation and provider online survey

##### 2.3.4.1 Prospective multi-center cohort study in module 3

Primary data will be collected at two different time points: t0, at the initial visit of the ED or an alternative care structure, and at t1, a 30-days follow-up. Primary data will be collected by means of a patient survey at t0 in the ED and waiting rooms. As this is an observational study, the routine course of treatment is not interrupted at any time and there are no safety concerns to consider regarding study procedure. Safety endpoints are assessed to evaluate the safety of the different study arms.

At t1, primary data collection will be conducted by online survey or telephone interview. Patient questionnaires for t0 and t1 are available in two languages: German and English. While validated translations for items were used whenever possible, the majority of instruments were translated using an adapted approach to the TRAPD method: This approach involved relying on two independently produced translations, which were created with the assistance of the KI-based translation service of DeepL™ (deepl.com), a bilingual review panel with expertise in both the subject area and research methods supported the translation process (28).

At both time points, t0 and t1, patient-reported outcome and experience measures (PROMs, PREMs) are collected, e.g., reasons for ED visit at t0, and satisfaction with care (ZUF-8) (29) and health system responsiveness at t1 (30). Patient-reported outcome and experience measures are complemented by measurements of baseline socio-demographics and—economics as well as navigational health literacy (NAV-HL) (31) at t0. Secondary data will be extracted, if applicable, from clinical records and from the hospital information systems and the outpatient care unit documentation system at t0. Information on resource use and costs at t0 as well as self-reported health service utilization at the 30-days follow-up (t1) are used for a health economic evaluation. In study arm 3, the assessment of patient urgency and level of care will be measured and documented using a validated assessment form for expert evaluation (32).

A supplementary part of module 3 is the development and validation of a standard item to measure reasons for low-acuity ED attendances based on international findings and on theoretical reference to the Behavioral Model of Health Care Utilization (33). See Table 1 for a schedule of enrolment, interventions and assessments in module 3.

**Table 1:** Schedule of enrollment, interventions, and assessments of module 3 of the NODE study.

	Study period			
	Enrollment	Data collection period		Close-out
TIMEPOINT	0	*t_0_ (Baseline)*	*t_1_ (30 days follow-up)*	*month 20 until 36*
ENROLLMENT module 3:				
Eligibility screening	X			
Written informed consent	X			
Survey participation		X	X	
Dissemination				X
ASSESSMENTS:				
Baseline variables	X	X		
Questionnaire		X	X	
Participant observation		X		
Urgency and care level assessement (study arm 3)		X		
Secondary data extraction		X	X	
DATA ANALYSES				X

t0 = baseline, t1 = follow up.

##### 2.3.4.2 Outcomes and measures

In module 3, primary and secondary outcomes will be collected, see Table 2.

**Table 2:** Overview of outcomes of module 3 of the NODE study.

Outcome	Concept	Instrument/parameter	Time point of measuring & sample size
Primary outcome			
	Effectiveness of the care model	Proportion of patients that are actually treated (study arm 1 and 2)—or could have been treated (study arm 3) in an alternative outpatient emergency care model instead of in the ED	t0 (*n* = 1,031)
Secondary outcomes			
	Efficiency of care model	Proportion of cases who are actually treated in the alternative care structure without being referred back to the ED.	t0 (*n* = 1,031)
	Process quality	Process times (e.g., duration of treatment, time until diagnostic measures are available/discharge), frequency of diagnostic procedures	t0 (*n* = 1,031) & t1 (*n* = 344)
	Patient safety	Adverse events: Mortality, unplanned (re-)admission to the ICU, unplanned inpatient admission, unplanned re-attendance at the ED within 30 days	t0 (*n* = 1,031) & t1 (*n* = 344)
	Patient satisfaction	ZUF-8 (29)	t1 (*n* = 344)
	Health-related quality of life	EQ-5D-5l (34)	t1 (*n* = 344)
	Costs and Cost-effectiveness	Costs calculated based on utilization of: hospital stays, outpatient visits, inpatient stays, medication/pharmaceuticals, adjuvants and devices, diagnostic and therapeutic measures, total costs in relation to primary outcomes	t1 (*n* = 344)
	Further clinical outcomes (analysis depending on the sufficient number of cases)	Length of inpatient stay, frequency of ICU stay, length of ICU stay at t0 and t1, if applicable.	t0 (*n* = 1,031) & t1 (*n* = 344)

ED, emergency department; EQ-5D-5l, 5-level EQ-5D version of the EuroQoL group questionnaire; ICU, intensive care unit; ZUF-8, “Fragebogen zur Messung der Patientenzufriedenheit” = Zurich patient satisfaction questionnaire. T0 = baseline, t1 = follow up. Study arm 1 = hospital-owned separate outpatient care model within the ED premises or on the hospital grounds; study arm 2 = urgent care practice of the Regional Association of SHI Physicians at the ED location; study arm 3 = ED with standard care model and prospective assessment of patient-care and urgency levels by ED doctors.

##### 2.3.4.3 Power calculation in module 3

The power calculation is based on the primary endpoint—effectiveness of the care model, which is operationalized as the proportion of patients that are treated or could have been treated (study arm 3) in an alternative care structure other than regular ED care: A total sample size of 1,031 patients was confirmed to be recruited among the six study centers. The primary endpoint will be collected based on documented patient flows by study personnel and from secondary data of the respective study centers. The final approach needs to be adapted to local data availability at the study sites. The assumptions on the frequencies of the primary endpoint do not refer to all ED patients, but to the population matching the inclusion and exclusion criteria of the study which is estimated at 50% of all ED patients, based on scientific literature (35, 36). We assume that different proportions of patients with clinically low-acuity consultation needs will be treated in the three different patient navigation models. Assumptions for power calculation purposes were derived from scientific literature (1–7, 9, 37–41) and expert opinion: Study arm 1) The hospital-owned separate outpatient care facility within the ED premises or on the hospital grounds is suspected to lead to a reduction of 30% of ED visits; Study arm 2) Approximately 50% of all ED cases are assumed to be treated by the urgent care practice of the Regional Association of SHI Physicians at the ED location; Study arm 3) The proportion of patients in standard care who could potentially be treated in an alternative care structure is estimated at 70%. Therefore, to detect differences between study arms with a power of 80% and an alpha error of 5%, a sample size of *n* = 31 patients per study arm is required. Despite the low number of participants to determine the difference between study arms, we still aim for a higher precision to estimate the frequencies of the primary endpoint in the study arms. A precision of 5% based on the binomial 95% confidence interval is assumed. This results in different sample sizes per arm:

Study arm 1: Assumed frequency of the primary endpoint 30%—*n* = 323
Study arm 2: Assumed frequency of the primary endpoint 50%—*n* = 385
Study arm 3: Expected incidence of primary endpoint 70%—*n* = 323

This gives a final sample size of *n* = 1,031. The aim is that approximately one third of the recruited patients (*n* = 344) will consent to and successfully complete a follow-up survey 30 days after their emergency department visit (t1). Equal numbers for t1 across all study arms enable comparisons for exploratory analyses of secondary endpoints. This estimate is based on the assumption that approximately 50% (*n* = 516) of the patients will agree to a follow-up interview, of which a further 17% (approximately *n* = 172 patients) will be unavailable or unable to complete the interview successfully for other reasons.

##### 2.3.4.4 Participant observation of patient treatment paths and organizational processes in module 3

Participant observations (*n*∼10–15) will explore and document treatment paths of patients in the different navigation models of three study arms. These observations will result in digital protocols and digital patient flow diagrams. The aim of the participant observations is the in-depth exploration of model differences, patients' expressed treatment experience, their treatment paths, behavior and navigation within the respective care settings as well as provider-patient interaction. Participant observations will capture the structural and processual differences within and between study arms and hereby provide context-sensitive data for triangulation with the qualitative interviews and the multi-center cohort study, particularly with regard to model performance in terms of efficiency and patient safety, patient satisfaction (29) and measures of navigational health literacy (31) as well as health system responsiveness (30).

##### 2.3.4.5 Provider online survey in module 3

An exploratory and anonymous quantitative online survey among a subgroup of health care providers working in the context of emergency care in Germany will be conducted, distributed by the professional networks of the authors. The survey aims to investigate which patient navigation models in emergency care and instruments are used at different locations in Germany and how providers assess the efficiency of these models.

##### 2.3.4.6 Data analysis plan of module 3

Descriptive analyses will be performed among the total patient cohort and within the study arms for patient characteristics, as well as for primary and secondary endpoints. Relative and absolute frequencies will be reported for categorical variables, whereas for quantitative variables mean and standard deviation will be reported in case of normal distribution, median and inter quartile ranges for not normally distributed variables. The chi-square test will be used to investigate differences between study arms for categorical variables, while an analysis of variance (ANOVA; in case of normal distribution) or Kruskal–Wallis-Test (in case of no normal distribution) will be used to detect differences between study arms for quantitative variables. The evaluation of the primary endpoint—effectiveness (i.e., proportion of patients who actually were or potentially could be treated in the alternative outpatient emergency care model) will be carried out using a chi-square test and binomial 95% confidence intervals. Furthermore, factors associated with primary and secondary endpoints will be investigated. For continuous endpoints, analysis of covariance (ANCOVA) will be applied while binary endpoints will be analyzed by logistic regression or Poisson regression analyses. Survival analyses will be performed using Cox regression and Kaplan–Meier analysis. In case of significant associations between socio-demographic variables and secondary outcomes, a stratified analysis will be conducted. Non-parametric methods are used depending on the distribution of variables. Generalized linear mixed models (GLMM) and generalized estimating equations (GEE) are used for longitudinal data. In these analyses, *p*-values are interpreted exploratively, not corrected for multiple testing, and appropriate effect size measures are used.

At t0, cost data from each participant will be mapped in standardized cost rates from the health care provider perspective. At t1, a cost-effectiveness analysis will be performed from the perspective of the German statutory health insurance to compare patient-reported health care utilization between the three study arms. Effects in terms of clinical endpoints (i.e., patient safety) will be compared with health care costs (utilization costs). Firstly, ANOVAs and/or if applicable ANCOVAs will be performed to identify significant cost differences between study arms. Patient characteristics may differ between the study arms. Thus, results are going to be adjusted with regard to possible confounders (e.g., age, triage level, comorbidities) to ensuring comparability. Secondly, average cost-effectiveness ratio (CER) and incremental cost-effectiveness ratio (ICER; comparing study arm 1 and 2 with standard emergency care of study arm 3) will be calculated to identify a dominant study arm in terms of cost-effectiveness. In a last and third step, bootstrapping can be conducted as sensitivity analysis to assess robustness of the results.

Qualitative content analysis will be applied to the digital observation protocols, following the analysis steps outlined in the data analysis plan for module 2 (25). In addition, the coding scheme for observation protocol analysis will be developed by two independent researchers using a random selection of protocols (10%–25% of the number of protocols) until there is acceptable overlap in coding (inter-coder reliability for qualitative content analysis; Krippendorff's *α* > 0.67), signifying that a validated scheme has been obtained (25, 42). The entire set of protocols is then coded. Quantitative data from the prospective multi-center cohort study is reported for individual observed patients in relation to the average of the entire survey population or specific subgroups to preserve anonymity. Results from the observation protocols are reported according to the standards for reporting qualitative research (27).

##### 2.3.4.7 Data management plan of module 3

Primary data from patient surveys will be entered into an electronic Case Report Form (eCRF) by patients at t0 and t1 (using a handheld tablet at t0 or private device at t1) and if applicable by study nurses (e.g., in case of assistance at to or telephone interviews at t1) using a handheld tablet or via desktop application. Secondary routine clinical data (e.g., triage category, pain scale, vital signs, diagnosis, timestamps, diagnostic and therapeutic procedures, in-patient stay and discharge from ED or from hospital) will be extracted from hospital records to an eCRF by study nurses. Doctors' assessments of patients' medical urgency and potential level of care, on which the primary endpoint in study arm 3 is based, is also entered into an eCRF by study nurses. All extracted data is pseudonymized and will allow subsequent data linkage with patient reported data from surveys.

Participant observation is documented in written, digitalized, and pseudonymized protocols and linked to pseudonymized primary and secondary data.

Upon request, anonymized data from the multi-center cohort study will be made available for secondary analysis for research purposes in justified cases.

#### 2.3.5 Module 4: data triangulation and recommendations for practice and policy

Data from modules 2 and 3 will be triangulated to advance and contextualize interpretations of the findings and to derive policy and practice recommendations from the findings. To this end, mixed-methods matrix (43) will be produced, combining results from qualitative and quantitative data collection on specific endpoints (e.g., patient satisfaction) and emerging themes. In an iterative and abductive process, the results will be related back to existing conceptual models [e.g., complex adaptive systems theory (44), macro-meso-micro model of organization-related health services research (45)] to help integrate the main findings. An existing model may be chosen or adapted.

Moreover, a symposium will be organized to communicate findings and lessons learned to national stakeholders, such as representatives of patient organisations, physician and hospital associations, policy makers or other researchers and experts in the field of emergency and acute medicine. Subsequently, in a structured group discussion, participants will develop implications for policy and practice, focusing on questions as:

– Which international patient navigation models in emergency care (module 1) are transferable to the German health care system?
– What optimizations are recommended for existing patient navigation models?
– What are the requirements for the reimbursement of navigation models from the payer perspective?

## 3 Discussion

To the best of our knowledge, this study will provide the first systematic and mixed-method approach to evaluate currently practiced navigation models of patient navigation in German acute and emergency care. Drawing on a health system perspective, it aims to provide a comprehensive analysis of the structure, processes, and performance of these models that offers insights both at the level of the individual hospital and within the broader health care system—an approach that is novel in the given context. The evaluation will incorporate both provider and patient perspectives, inquiring aspects such as workflow impact and workload relief on the provider side, as well as patient satisfaction and navigation-related capabilities on the patient side. In doing so, the study contributes to understanding how social factors and individual attributes—such as health literacy, gender, migration background—may influence patients' experience of navigation models.

Our findings on aspects such as model efficiency, patient safety, patient satisfaction, and cost-effectiveness may support ED managers and policymakers in adapting or implementing patient navigation models, particularly with regard to their format (e.g., inter-sectoral collaboration, required professional training and roles, and the potential for standardizing initial assessment procedures). The involvement of relevant stakeholders in this study (design and evaluation strategy) will ease the implementation process into policy and practice, hence, the development process of novel or adapted navigation models in German emergency care can be facilitated (46, 47).

However, a potential limitation of this study is that structural changes due to health policy reforms concerning alternative emergency care models (i.e., SHI urgent care, partner practices) in the model region of Berlin could and did occur during the study period, necessitating an adjustment of the study arms. The originally planned third study arm “partner practice model” (a collaborative model between EDs and outpatient practices in which ED personnel arranges appointments with collaborating outpatient practices for patients with low-acuity consultation needs) could not be implemented due to the delayed introduction of this model at sites in Berlin by the Association of SHI Physicians as well as a change in the legal basis for such forms of care. Instead of the partner practice model, the standard care in the ED is now being examined in study arm 3. As a result of the study adaptation, an updated calculation and adjustment of the planned recruitment numbers was necessary. Therefore, study protocol publication occurred after initiation of data collection.

Even at the recruitment stage, the study potentially can be affected by further changes., e.g., reduction of the opening hours or discontinuation of care provision models. This would require additional adjustments in the study design and planned recruitment numbers, resulting potentially in an insufficient number of recruited patients for module 3. However, recruitment of participants be expanded by additional study centres and an extended duration of the recruitment period.

A final limitation may be that the data from patients and health care providers are limited to the city of Berlin. To address the methodological challenge of external validity, we will triangulate our findings with the results of existing pilot studies conducted in similar contexts and the findings of the provider online survey in Module 3. We will then discuss our interpretations in a workshop involving international experts and practitioners. Potential limitations in ecological validity—such as the inability of observational data to fully capture timing, social dynamics, or implicit decision-making—will be addressed through triangulation with focus group and interview data, as well as through external expert feedback and findings from the existing literature. In addition, we actively incorporate reflexivity throughout the research process. This includes presenting preliminary findings in academic conferences and workshops, allowing for critical external feedback. These reflexivity loops serve to identify potential biases and strengthen the methodological robustness of the study. Further research will be needed to assess the representativeness of the results for Germany as a whole.
